# Effects of storage conditions and digestion time on DNA amplification of biting midge (*Culicoides*) blood meals

**DOI:** 10.1186/s13071-022-05607-x

**Published:** 2023-01-13

**Authors:** Ben Bellekom, Abigail Bailey, Marion England, Zoe Langlands, Owen T. Lewis, Talya D. Hackett

**Affiliations:** 1 Department of Biology, University of Oxford, 11a Mansfield Road, Oxford, OX1 3SZ UK; 2 The Pirbright Institute, Ash Road, Pirbright, Surrey, GU24 0NF UK

**Keywords:** *Culicoides*, DNA degradation, Metabarcoding, Blood meal, Biting diptera, DNA digestion

## Abstract

**Background:**

Molecular analysis of blood meals is increasingly used to identify the hosts of biting insects such as midges and mosquitoes. Successful host identification depends on the availability of sufficient host DNA template for PCR amplification, making it important to understand how amplification success changes under different storage conditions and with different durations of blood meal digestion within the insect gut before being placed into the storage medium.

**Method:**

We characterised and compared the digestion profile of two species of *Culicoides* over a 96-h period using a novel set of general vertebrate primers targeting the 16S rRNA gene. A set number of individuals from each species were killed over 13 time points post-blood feeding and preserved in 95% ethanol. Samples were stored either at ambient room temperature or in a − 20 °C freezer to examine the effect of storage condition on the PCR amplification success of host DNA.

**Results:**

We found that amplification success across the 96-h sampling period post-feeding was reduced from 96 to 6% and 96% to 14% for *Culicoides nubeculosus* and *Culicoides sonorensis*, respectively. We found no effect of storage condition on PCR amplification success, and storage in 95% ethanol was sufficient to maintain high rates of amplifiable host DNA for at least 9 months, even at room temperature.

**Conclusions:**

These findings highlight the limited time frame during which an individual may contain amplifiable host DNA and demonstrate the importance of timely sample capture and processing post-blood feeding. Moreover, storage in 95% ethanol alone is sufficient to limit host DNA degradation. These results are relevant to the design of studies investigating the biting behaviour and disease transmission potential of *Culicoides* and other biting Diptera*.*

**Graphical Abstract:**

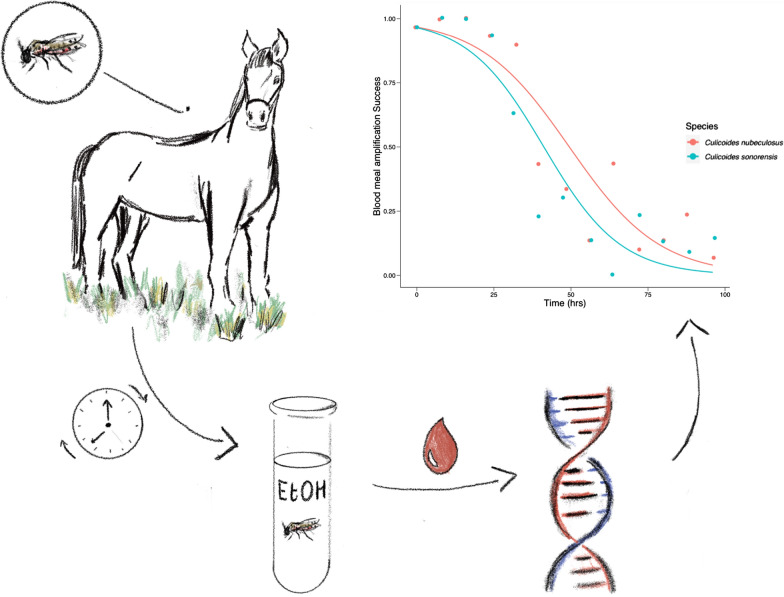

**Supplementary Information:**

The online version contains supplementary material available at 10.1186/s13071-022-05607-x.

## Introduction

Biting (haematophagous) Diptera are vectors of a wide range of pathogens that cause disease in humans as well as domesticated and wild animals. Transmission dynamics of these diseases are, in part, mediated by the host-biting preferences of the vectors [1]. Thus, data on vertebrate blood meal sources and host preferences can provide valuable information for understanding disease transmission [2], including transmission across the livestock and wildlife interface. Blood meal data can also be used to populate ecological interaction networks, helping to identify transmission pathways and reservoirs of infection and informing control strategies [3].

In the past, host blood meals were identified using serological methods which have now been largely superseded by molecular methods, diagnostic PCR, DNA barcoding and, most recently, metabarcoding [2, 3]. These methods rely on extraction of DNA from the blood meal and PCR amplification of a taxonomically informative fragment of host DNA [1] followed by sequencing of the PCR product or diagnostic PCR using primers specific to a species or set of species. From as little as US $2/sample at 2022 prices, barcoding is now a relatively low-cost method for sequencing a single gene, but it is unsuitable for mixtures of DNA from multiple species. Metabarcoding coupled with next generation sequencing allows many sequences to be read in parallel, facilitating the analysis of multiple gene loci and individual samples and the simultaneous identification of species within mixed samples such as gut contents [4] and blood meals [1]. Whilst sequencing costs have dramatically fallen, metabarcoding remains more expensive than targeted DNA barcoding. Price per individual sample may vary greatly depending on the sequencing kit and platform used, the number of samples being processed and the target read depth.

DNA degradation by digestion enzymes will reduce the success of PCR amplification, jeopardising successful blood meal diagnosis [2, 5–7]. In mosquitoes (Culicidae), digestion occurs rapidly, with host DNA undetectable within 32–72 h [5, 8]. However, rates of DNA degradation attributable to digestion are likely to vary among haematophagous taxa, and equivalent data for other biting Diptera families of medical, veterinary and epidemiological importance are lacking. Prompt preservation of blood-fed biting Diptera to halt digestive processes can increase the success of PCR amplification [9]. Preservation methods include storage in ethanol [10], desiccation [11], transferring blood meals onto filter paper [12] and cryopreservation [13, 14]. However, field constraints and institutional limitations often limit the use of − 20 °C and ultra-low (− 80 °C) freezers and collected samples may be left for extended periods at room temperature, especially during transport. It is therefore of considerable practical interest to understand how amplification success varies for samples stored under different conditions.

Here we examine the effect of digestion time and the impact of two storage conditions (ambient room temperature and a − 20 °C freezer) on the PCR amplification success of host DNA in blood meals from two species of *Culicoides*. Biting midges in this genus have a wide distribution globally, bite a broad range of host species [10] and are vectors of several pathogens of ﻿veterinary and medical significance [10, 15]. We used PCR amplification success to confirm the presence of sufficient quantities of non-degraded host template DNA to allow for further molecular analysis and host identification. In parallel, we assess the suitability of a novel 16S rRNA general vertebrate primer set for Diptera blood meal identification.

## Methods

We experimentally examined the effect of blood meal digestion time and storage method on the PCR amplification success of host DNA in two species of biting midges (*Culicoides nubeculosus* and *C. sonorensis*) using novel vertebrate PCR primers. Individuals from each species were fed fresh horse blood, killed and preserved in 95% ethanol at 13 specific time points, ranging from freshly fed to 96 h post-feeding, in 8-h intervals. At each time point, 30 individuals from each species were knocked down by freezing and stored in ethanol to minimise DNA degradation. An additional 10 individuals at time point 0 were collected to investigate long-term storage effects, thus resulting in 40 individuals at time point 0. In total, 800 midges were collected for blood meal analysis (Table 1).

**Table 1:** The number of individuals (and successful amplifications) for both species of *Culicoides* that underwent PCR amplification by time point (h) and storage method

Time point (h)	*Culicoides nubeculosus*	*Culicoides nubeculosus*	*Culicoides sonorensis*	*Culicoides sonorensis*
	Ambient	− 20 °C Freezer	Ambient	− 20 °C Freezer
0	20 (20)	20 (19)	20 (19)	20 (20)
8	15 (15)	15 (15)	15 (15)	15 (15)
16	15 (15)	15 (15)	15 (15)	15 (15)
24	15 (14)	15 (14)	15 (14)	15 (14)
32	15 (14)	15 (13)	15 (10)	15 (9)
40	15 (4)	15 (9)	15 (4)	15 (3)
48	15 (4)	15 (6)	15 (4)	15 (5)
56	15 (2)	15 (2)	15 (3)	15 (1)
64	15 (9)	15 (4)	13 (0)	12 (0)
72	15 (2)	15 (1)	15 (3)	15 (4)
80	15 (3)	15 (1)	8 (0)	7 (2)
88	15 (4)	15 (3)	12 (2)	10 (0)
96	15 (0)	15 (2)	7 (1)	7 (1)

To examine the effect of storage method, 15 samples from each species at every time point were preserved in 95% ethanol and stored in a − 20 °C freezer, whilst the remaining 15 were stored in ethanol and left at room temperature (22–24 °C). To minimise biases, samples were extracted and underwent PCR amplification in a randomised order across all time points and storage methods. To examine the impact of long-term storage on DNA integrity, two sets of five individuals of each species from the first time point were preserved in ethanol and stored under the two temperature conditions (− 20 °C freezer and at ambient room temperature), respectively, and left for 9 months before extraction and amplification. Extraction and amplification of all samples, excluding the long-term storage sets, took place across a 3-month period following sample preparation.

### Midge rearing

*Culicoides sonorensis* and *C. nubeculosus* specimens were obtained from lines maintained in existing closed colonies at the Pirbright Institute. The *C. nubeculosus* colony was established in 1969 from pupae collected in Hertfordshire, UK. The *C. sonorensis* colony was established from eggs provided by Dr. H. Jones who initiated a laboratory colony in Colorado in 1957. The colonies were maintained following previously developed protocols [16] with adult females fed on commercially supplied horse blood (TCS Biosciences, UK) using a Hemotek blood feeder (Hemotek, UK). Midges were not sugar fed during the 96-h period as a trial conducted prior to the commencement of the study indicated that unfed midges survived beyond the 96-h period (unpublished data). Furthermore, this most accurately represented the behaviour of females in the field following feeding, whereby they rest until oogenesis is complete and then find a suitable habitat to lay [17].

### Blood meal analysis

DNA was extracted from individual blood meals using a Qiagen DNeasy Blood and Tissue kit, using the standard protocol with the following minor alterations. Prior to lysis, individuals were homogenised using an MP Biomedicals FastPrep-24 5G homogeniser to release the blood meal from the abdomen. Prior to homogenisation, a single sterile 2.3-mm zirconia/silica agitating ball was placed in each 1.5-ml microcentrifuge tube with 180 µl of Buffer ATL. To minimise Buffer ATL foaming, which may reduce the homogenisation efficiency, 2 µl of Reagent DX was added to each microcentrifuge tube. To increase final DNA concentration, prior to elution, 60 µl of Buffer AE was pipetted onto the Dneasy spin column membrane and allowed to incubate at room temperature for 5 min. Final DNA concentration for all samples was quantified using a Qubit 3.0 fluorometer.

In the absence of a priori host assumptions and the potential presence of mixed blood meals (derived from different host species), the PCR amplification of host genomic DNA from blood meals of wild-caught *Culicoides* and other haematophagous Diptera requires the use of general vertebrate primers [18]. To facilitate the identification of horse-derived blood meals used in this study and to determine the likelihood of successful amplification in future blood meal metabarcoding studies, we designed primers intended to amplify vertebrate templates on the 16S rRNA gene, whilst excluding invertebrate templates. We downloaded 128 mitogenomes belonging to the classes Aves and Mammalia from the NCBI Genbank database. As these primers are designed for blood meal analysis of a wide range of biting Diptera, we also downloaded five biting Diptera mitogenomes (*Anopheles gambiae, Aedes albopictus, Aedes aegypti, Culicoides arakawae* and *Culicoides imicola*). The sequences were MAFFT aligned using the bioinformatics software Geneious Prime (Biomatters Limited, New Zealand). Where DNA is highly degraded, primer pairs targeting short amplicons are preferable. Consequently, this alignment was used to identify two potential 18–22-bp primer binding sites that contained primer-invertebrate annealing site mismatches, which produced a 200-base pair (bp) amplicon. Initial binding site selection was informed by previous work [19]. To ensure binding across all mammal and bird mitogenomes, two degenerate bases were inserted into the forward primer to account for single base mismatches. Primer protocol selection and validation were conducted using gradient PCRs with a range of known mammalian and avian DNA templates (Additional file 1: Text S1.). As the PCR primers were designed for use with a wide range of biting Diptera species, examination of blood meal amplification success and identification of non-specific binding was conducted on genomic DNA from blood fed and unfed *C. sonorensis* and *An. gambiae*. I. Fed and unfed *C. sonorensis* were obtained from the colonies at the Pirbright Institute. *Anopheles gambiae* individuals were wild caught and stored in 95% ethanol at field sites in Burkina Faso as part of ongoing research by members of the Target Malaria research consortium (see Acknowledgements). The PCR product of blood-fed *C. sonorensis* and *An. gambiae* were Sanger sequenced (Source Bioscience) to confirm successful target amplification.

The presence of sufficient concentrations of non-degraded host DNA template for PCR amplification was assessed using end-point PCR and our novel primer set, 16smbF (5′-GGT TGG GGY GAC CTY GGA-3′) and 16sbbR (5′--CTG ATC CAA CAT CGA GGT CGT A-3′-). PCR amplification was carried out in 25 μl reactions that contained 12.5 μl HotStarTaq Master mix (Qiagen, Germany), 10 μM of each primer, 8.5 μl H_2_O and 2 μl DNA template. The PCR protocol consisted of an initial denaturation step of 15 min at 95 °C followed by 35 cycles of 94 °C for 45 s, 58 °C for 45 s and 72 °C for 30 s, followed by a final extension step of 72 °C for 10 min. Two negative controls, containing nuclease-free water (ThermoFisher Scientific, USA), were included in every set of reactions to monitor for contamination.

PCR products were electrophoresed and visualised on a gel red (SYBR™ Safe, ThermoFisher Scientific, USA) stained 2% agarose gel. The presence of a band of the expected amplicon size was taken as a positive result, indicating that sufficient host DNA remained viable for blood meal identification. To confirm successful target amplification, a subset of PCR products from across the range of time steps that yielded bands were Sanger sequenced (Source Bioscience, England) and the sequence’s origin was identified using the basic alignment search tool (BLAST).

### Statistical analysis

We modelled the effect of digestion time, storage method and species on a binary measure of amplification success (1 or 0) using a multivariate binomial logistic regression. Model fit was evaluated with a likelihood ratio test and the significance of the overall effect of each variable was determined using the chi-squared statistic [5]. Data were analysed using R (version 4.1.2) and visualised using the package *ggplot2* [20].

## Results

Overall, 759 midge samples were used in this study. Some midges died during the later time steps (64 h post-feeding onwards), so four time steps had fewer than the planned 15 *C. sonorensis* individuals (Table 1).

**Fig. 1:**
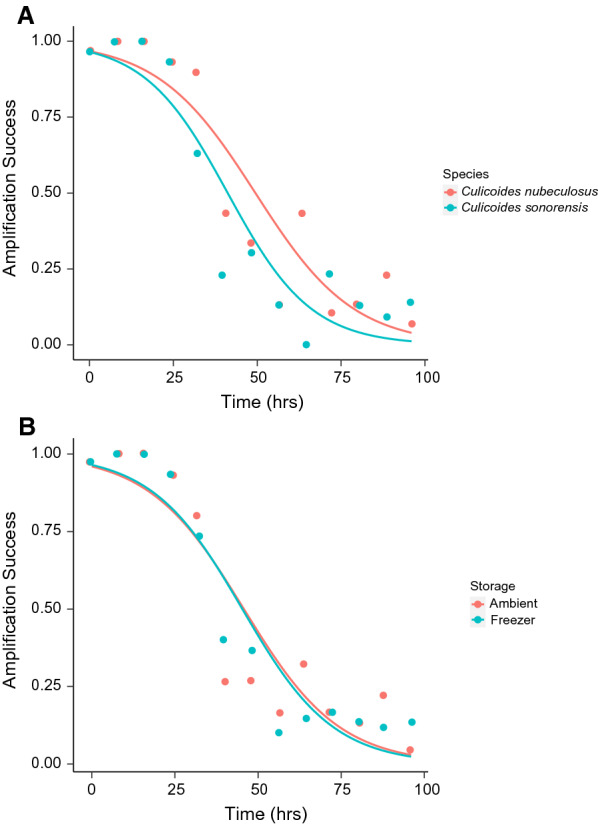
(**A**) PCR amplification success with time by *Culicoides* species. Red and blue points represent the amplification success of *Culicoides nubeculosus* and *C. sonorensis*, respectively, at a given time point. (**B**) PCR amplification success with time by storage method. The red and blue points represent amplification success of ambient and − 20 °C freezer, conditions respectively, at a given time point

Digestion time significantly decreased amplification success of host DNA (*X*^2^ = 402.48, df = 1, *P* < 0.001), with amplification success approaching 6% 96 h post-feeding (Fig. 1A). We found no significant effect of storage method on amplification success (*X*^2^ = 0.024, df = 1, *P* = 0.875; Fig. 1B). Amplification success was significantly higher for *Culicoides nubeculosus* than for *C. sonorensis* (*X*^2^ = 8.318, df = 1, *P* = 0.004; Fig. 1). All 20 long-term individuals retained amplifiable host DNA following the 9-month storage period.

## Discussion

We found that the duration of blood meal digestion, but not the storage condition (ambient or − 20 °C freezer), had a significant impact on the success of host DNA PCR amplification and thus the likelihood of subsequent host identification. Whilst four time steps post 64 h had fewer *C. sonorensis* individuals than planned, amplification during this period was limited in both species and the reduction in sample size is unlikely to have impacted the findings significantly.

Increased post-feeding digestion time significantly reduced amplification success of blood meals from both colony *Culicoides* species used in this study, with a rapid decrease after the 32-h time step. Similar results have been documented in mosquitoes [7], suggesting that rates of DNA degradation in digested blood meals can be similar in hematophagous taxa that differ markedly in blood meal size. Consistency in the digestion profiles of these taxa may reflect similar production patterns of digestive enzymes such as late trypsin, a protease responsible for the endoproteolytic cleavage of protein in the blood meal [21–23].

Our findings highlight the limited time frame over which samples are usable for blood meal analysis and the importance of timely sample capture and processing and may partially explain variations in *Culicoides* blood meal amplification success in previous studies [10, 24]. Whilst it was possible to amplify host DNA successfully at the later time steps, success was significantly reduced and may bias later analyses, such as examination of host preferences and species roles in networks. Selection of trapping methods and sampling protocols should account for this to maximise the proportion of amplifiable blood meals. Specifically, we show that storage in 95% ethanol limits blood meal degradation of freshly fed biting Diptera even after prolonged periods of storage. Furthermore, individuals should ideally be killed as soon as possible after feeding to halt digestive processes, using trapping methods that catch individuals directly into ethanol. Traps commonly used for *Culicoides*, such as OVI (Onderstepoort Veterinary Institute) light traps and CDC (Centers for Disease Control) light traps, use ethanol-filled collection chambers. However, mosquitoes are often collected with designs such as the BG Sentinel Trap, where collections are kept dry to facilitate morphological identification of specimens. Therefore, if the primary goal is to identify host blood meals, it may be advisable to modify these traps to include an ethanol-filled chamber, although this may necessitate molecular identification of the sampled insect if immersion in liquid causes damage to scales used for morphology-based identifications.

Halting digestion and minimising degradation of host DNA post-capture is of particular importance for downstream blood meal analysis. We found no significant difference in amplification success between samples stored in 95% ethanol in a − 20 °C freezer versus at room temperature, suggesting that storage in 95% ethanol is sufficient to limit host DNA degradation in *Culicoides*. These findings are consistent with previous work, which suggested that ethanol alone is sufficient to maintain host DNA integrity in mosquito blood meals [5, 25]. The limited effect of storage at ambient temperatures is relevant for the planning and logistics of field work. Collection of *Culicoides* and other biting Diptera often necessitates sampling in remote locations without access to freezers; our results provide reassurance that, given proper processing, samples can safely be stored and transported at ambient temperatures for extended periods without impacting amplification success. The range of ambient temperatures used in our study reflects those in temperate regions, where monitoring of *Culicoides* species and limiting the spread of *Culicoides*-borne diseases is a major concern [24, 26–30]. Ethanol has also been shown to be an effective short- to medium-term mosquito blood meal storage method under higher temperature tropical conditions [5]. This suggests that our results might also apply to *Culicoides* samples stored under ambient conditions in the tropics, though further work is required to confirm this; there is potentially an upper temperature limit where 95% ethanol does not sufficiently halt DNA degradation in blood meals.

We found slightly greater amplification success for blood meals of *C. nubeculosus* compared with *C. sonorensis*. These *Culicoides* species are of similar size, making it unlikely that blood meal size was a factor [31]. Differences between the species were most apparent at a time estimated to correspond to stage 3 of *Culicoides* digestion [32], during which secretory granules associated with the production of digestive enzymes are formed. Thus, the differences in amplification success between the species may reflect differences in granule and digestive enzyme production. Alternatively, the higher mortality of *C. sonorensis* during the later time steps (64 h onwards) and the differing amplification success for the two species may reflect minor differences in the abiotic conditions under which the individuals were reared or slight differences in life history traits. For example, minor variations in ambient temperature can impact longevity [33] and rates of blood meal digestion [7].

General vertebrate PCR primers, when combined with high-throughput sequencing, allow identification of the range of host DNA template contained in mixed blood meals (metabarcoding). Moreover, general primers that target small amplicons are advantageous in blood meal analysis because of the high degree of DNA degradation resulting in fragmentation of the DNA template [1]. We identified a 200-bp region of the 16 s gene that is suitable for the interrogation of blood meals from a wide range of biting Diptera whilst avoiding co-amplification of Dipteran DNA. Our primers and PCR protocol were effective with highly degraded DNA, successfully amplifying host templates during later time steps.

## Conclusions

Based on the findings of this work, we propose the following recommendations. We suggest the use of the primers and the PCR protocol described here to complement existing primer sets in future biting Diptera blood meal analysis. Moreover, because of the efficiency and versatility of ethanol for maintaining blood meal DNA integrity and the need to halt digestive processes to maximise amplification success, we recommend the use of trapping methods that contain an ethanol-filled collection device, though potential ethanol evaporation should be accounted for by regular trap emptying and replenishment of ethanol. During field sampling of blood-fed biting Diptera, digestion of the blood meal may occur in the time between initial feeding and successful trapping. However, the placement of traps near potential sources of blood meals to limit digestion time may be unadvisable, depending on the overall aims of the trapping, as this will bias any host selection data [3]. These findings are relevant to future work that aims to investigate the biting behaviour and disease transmission potential of *Culicoides* (and likely other small haematophagous Diptera) through blood meal analysis.

## Supplementary Information

**Additional file 1: Text S1.** Primer protocol selection method.

## Data Availability

The datasets used and/or analysed during the current study are available from the corresponding author on reasonable request.
