# Supplementary material for: Effects of storage conditions and digestion time on DNA amplification of biting midge (Culicoides) blood meals
Source: Parasit Vectors. 2023 Jan 13;16:13. doi: 10.1186/s13071-022-05607-x (PMC9837887; doi:10.1186/s13071-022-05607-x)
Supplement: Supplementary file 1 — Additional file 1: Text S1. Primer protocol selection method. [file 13071_2022_5607_MOESM1_ESM.pdf]

Additional file 1: Text S1. Primer protocol selection method

To examine primer annealing temperature and binding success, DNA was extracted from seven meats, covering a range of mammalian and avian families: beef, venison, pork, goat, duck, chicken and pigeon. Additionally, DNA from a male mosquito and blood fed and unfed *C. sonorensis* and *A. gambiae* was extracted (see methods for extraction and PCR chemistry).

To determine ideal annealing temperature for the primers, a gradient PCR was carried out for each meat. Samples from each meat with similar starting DNA concentrations were selected for use in gradient PCRs. An initial gradient PCR with a temperature range of 60-70°C was conducted using beef samples. Following examination of the PCR success, all subsequent gradient PCRs were carried out with a temperature range of 58-65°C.

Following selection of an ideal annealing temperature, a PCR was repeated for each meat extract using the same PCR reaction volumes and refined PCR protocols: 15 min at 95°C followed by 35 cycles of 94°C for 45 sec, 58°C for 45 sec, and 72°C for 30 sec followed by a final extension step of 72°C for 10 min.
